# Open reduction and internal fixation versus casting for highly comminuted and intra-articular fractures of the distal radius (ORCHID): protocol for a randomized clinical multi-center trial

**DOI:** 10.1186/1745-6215-12-84

**Published:** 2011-03-22

**Authors:** Christoph Bartl, Dirk Stengel, Thomas Bruckner, Inga Rossion, Steffen Luntz, Christoph Seiler, Florian Gebhard

**Affiliations:** 1 Department of Orthopaedic Trauma Surgery, University of Ulm, Steinhoevelstr. 9, 89075 Ulm, Gemany; 2 Center for Clinical Research, Dept of Trauma and Orthopaedic Surgery, Unfallkrankenhaus Berlin, Warener Str. 7, 12683 Berlin, Germany; 3 Abteilung Medizinische Biometrie & SDGC, INF 305, D-69120 Heidelberg, Germany; 4 SDGC - Studienzentrum der Deutschen Gesellschaft für Chirurgie, Chirurgische Universitätsklinik, Im Neuenheimer Feld 110, 69120 Heidelberg, Germany; 5 Koordinierungszentrum für Klinische Studien (KKS), am Universitätsklinikum Heidelberg, Voßstraße 2/Gebäude 4410, 69115 Heidelberg, Germany

## Abstract

**Background:**

Fractures of the distal radius represent the most common fracture in elderly patients, and often indicate the onset of symptomatic osteoporosis. A variety of treatment options is available, including closed reduction and plaster casting, K-wire-stabilization, external fixation and open reduction and internal fixation (ORIF) with volar locked plating. The latter is widely promoted by clinicians and hardware manufacturers. Closed reduction and cast stabilization for six weeks is a simple, convenient, and ubiquitously available intervention. In contrast, ORIF requires hospitalization, but allows for functional rehabilitation.

Given the lack of randomized controlled trials, it remains unclear whether ORIF leads to better functional outcomes one year after injury than closed reduction and casting.

**Methods/Design:**

ORCHID (Open reduction and internal fixation versus casting for highly comminuted intra-articular fractures of the distal radius) is a pragmatic, randomized, multi-center, clinical trial with two parallel treatment arms. It is planned to include 504 patients in 15 participating centers throughout Germany over a three-year period. Patients are allocated by a central web-based randomization tool.

The primary objective is to determine differences in the Short Form 36 (SF-36) Physical Component Score (PCS) between volar locked plating and closed reduction and casting of intraarticular, comminuted distal radius fractures in patients > 65 years of age one year after the fracture. Secondary outcomes include differences in other SF-36 dimensions, the EuroQol-5D questionnaire, the Disability of the Arm, Shoulder, and Hand (DASH) instrument. Also, the range of motion in the affected wrist, activities of daily living, complications (including secondary ORIF and revision surgery), as well as serious adverse events will be assessed. Data obtained during the trial will be used for later health-economic evaluations. The trial architecture involves a central statistical unit, an independent monitoring institute, and a data safety monitoring board. Following approval by the institutional review boards of all participating centers, conduct and reporting will strictly adhere to national and international rules, regulations, and recommendations (e.g., Good Clinical Practice, data safety laws, and EQUATOR/CONSORT proposals)

**Discussion:**

To our knowledge, ORCHID is the first multicenter RCT designed to assess quality of life and functional outcomes following operative treatment compared to conservative treatment of complex, intra-articular fractures of the distal radius in elderly patients. The results are expected to influence future treatment recommendations and policies on an international level.

**Trial registration:**

ISRCTN: ISRCTN76120052

Registration date: 31.07.2008; Randomization of first patient: 15.09.2008

## Background

Distal radial fractures represent the most common injuries in humans and make up a considerable workload in orthopaedic and surgical departments worldwide. In population-based investigations, incidence rates vary from 5.7 to 124.6 per 10,000 person-years[1-21]. Together with fractures of the proximal humerus, vertebral bodies and the proximal femur, fractures of the distal radius typically mark the onset of symptomatic osteoporosis[22-24].

Established treatment options comprise closed reduction and cast stabilization, external fixation, and open reduction with internal plate fixation (ORIF). The first two options may be combined with percutaneous K-wire pinning.

According to a Cochrane Review of randomized controlled trials (RCT) and later clinical investigations[25-27], the most effective, efficient, and safe method of treating complex intraarticular wrist fractures remains unclear.

To date, a single RCT has compared ORIF to casting (19 and 23 patients, respectively) [28], suggesting a higher likelihood of excellent function with ORIF than with closed reduction (risk ratio [RR] 0.69, 95% confidence interval [CI] 0.48 - 1.00). The same trial also compared ORIF to external fixation (19 and 18 patients), without any difference in the rates of excellent function (RR 0.95, 95% CI 0.59 - 1.52). Two trials in 2004 and 2005 suggest better functional outcomes with external fixation compared to internal fixation with dorsal Pi-plates [29-31].

In recent years, angle-stable, volar locking plates have been propagated and enthusiastically used for surgical fixation of distal radial fractures, especially in the osteoporotic bone. The underlying biomechanical principle of angle-stable locked plating is that uni-cortical, threaded screws fixed in the screw hole of the plate ("internal fixator") reduce shear forces, thereby preventing loosening of the surgical construct. However the available clinical evidence in favor of this principle is limited to case series of moderate to poor quality [32-38].

The currently largest reported series of 114 patients also signals high complication rates with volar locking plate fixation of complex wrist fractures. Although functional outcomes after one year were reasonable, 31 patients (27%, 95% CI 19 - 36%) faced complications, mainly tendon irritation, carpal tunnel syndrome, and complex regional pain syndrome (CRPS) [39].

Regardless of these critical reports, volar locked plating has emerged as the surgical standard of care for managing distal radial fractures in Europe and the US[40].

## Study Rationale

Closed reduction and plaster casting is an ubiquitous, simple, safe, and cost-effective intervention to treat distal radial fractures. Casting may be applied in an outpatient setting but causes the discomfort of four to six weeks of immobilization of the wrist. In contrast, ORIF needs a hospital environment, well equipped operating rooms, and costly surgical material, but allows for early mobilization without cast immobilization.

**Figure 1:**
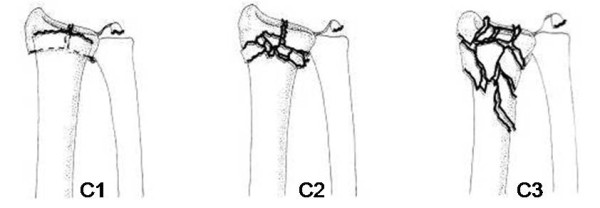
**AO/ASIF-classification**. AO/ASIF-classification of intraarticular distal radius fractures (C1,C2,C3)

Comminuted intraarticular wrist fractures (type 23 C1, C2, C3 according to the common classification rules of the AO Foundation [AO/ASIF] and the Orthopaedic Trauma Association [OTA]) destroy cartilage layers and compromise joint integrity (Figure 1). Biological fracture treatment aims at restoring the axis and length of the injured bone, reconstructing the articular surface, and achieving a stable construct that allows for early mobilization and weight bearing. However, excessive reconstruction with technically demanding and time consuming fixation of multiple small fragments may produce more good than harm, especially in the osteoporotic bone, osteoarthritis of the radio-carpal and adjacent joints, and poor skin and soft tissue conditions.

Thus, the obvious biomechanical advantages with internal fixation (anatomic reposition and stable fixation) must be balanced against its potential biological disadvantages (violation of the skin-soft tissue-bone-compound).

Given the high costs of this surgical intervention and taking into account its so far unproven benefits for elderly patients, there is an urgent need for a large, multi-center RCT comparing operative with non-operative treatment. This trial should clarify whether the anticipated benefits with ORIF hold against its invasiveness, potential complications, and costs.

## Primary Hypothesis

Because of previous studies and international trends in health services research, the investigators consider the Short Form 36 (SF-36) Physical Component Score (PCS) as the key outcome of this trial. They assume that, based on data from other studies, a difference of 8 points (on a scale of 50) with a pooled standard deviation (SD) of 3 (leading to a standardized mean difference of 8/3 = 2.67) between the experimental and the control intervention is of both clinical and health-political interest. As there are still no generally accepted 'minimum clinically important differences' (MCID) of the SF36-PCS in patients with fractures of the long bones, the predefined MCID was mainly based on the widely used SEM criterion [41]

*The primary hypothesis is that, after a follow-up of one year, there is a clinically relevant standardized mean difference of 2.67 points in the SF-36-PCS between surgical and non-surgical treatment of intra-articular fractures of the distal radius in elderly patients*.

## Methods And Design

### Design

The ORCHID (Open Reduction and internal fixation versus Cast immobilization for Highly comminuted, Intra-articular fractures of the Distal radius) trial is a pragmatic, multi-center RCT that aims at evaluating differences in patient-centered outcomes between two major options (i.e., volar locked plating versus closed reduction and cast stabilization) for treating wrist fractures in an elderly population. 600 patients will be recruited at 15 hospitals of various levels of care and associated private practices throughout Germany. It is expected that 150 patients are recruited during the first year after the initiation of all centers, followed by recruitment of 300 and 150 patients in the subsequent years.

### Eligibility criteria

Men and women aged 65 years or older with an isolated, unilateral, closed, comminuted, intra-articular fracture of the distal radius (AO/OTA 23 C1, C2, C3) according to the judgment of the surgeon on call are enrolled onto the trial.

To avoid misclassification of exposure, all radiographs will be evaluated centrally and independently by an orthopaedic surgeon and a radiologist. In case of disagreement there will be a consensus evaluation.

Only patients with a maximum interval of one week between injury and intervention must be included. No specific surgical pre-treatment (i.e., K-wiring, external fixation) is allowed. Patients must be able to understand the meaning of the trial and its consequences. Written informed consent or verbal agreement in presence of a witness in case of a fracture of the dominant hand and inability to write is mandatory for trial inclusion.

Patients will be excluded if the responsible surgeon requests surgical treatment, especially in case of open fractures or other situations in which the principle of therapeutic uncertainty (equipoise) is violated.

Also, patients with a high risk of anaesthesiology problems (i.e., ASA risk score > 3), acute infection, extreme obesity (i.e., BMI > 35 kg/m²), mental illness or low expected compliance will be excluded from trial participation. If patients issue a certain treatment preference, they will be excluded as well.

### Randomization

After having agreed with trial participation, patients will be randomized by the responsible surgeon to one of the treatment arms, using a web-based randomization tool (http://www.randomizer.at). Randomization is performed by blocks with stratification for participating centers.

### Interventions

Patients must be included only if there was no specific (i.e., physician-performed or -prescribed) pretreatment within one week after a traumatic exposure to the wrist.

**Figure 2:**
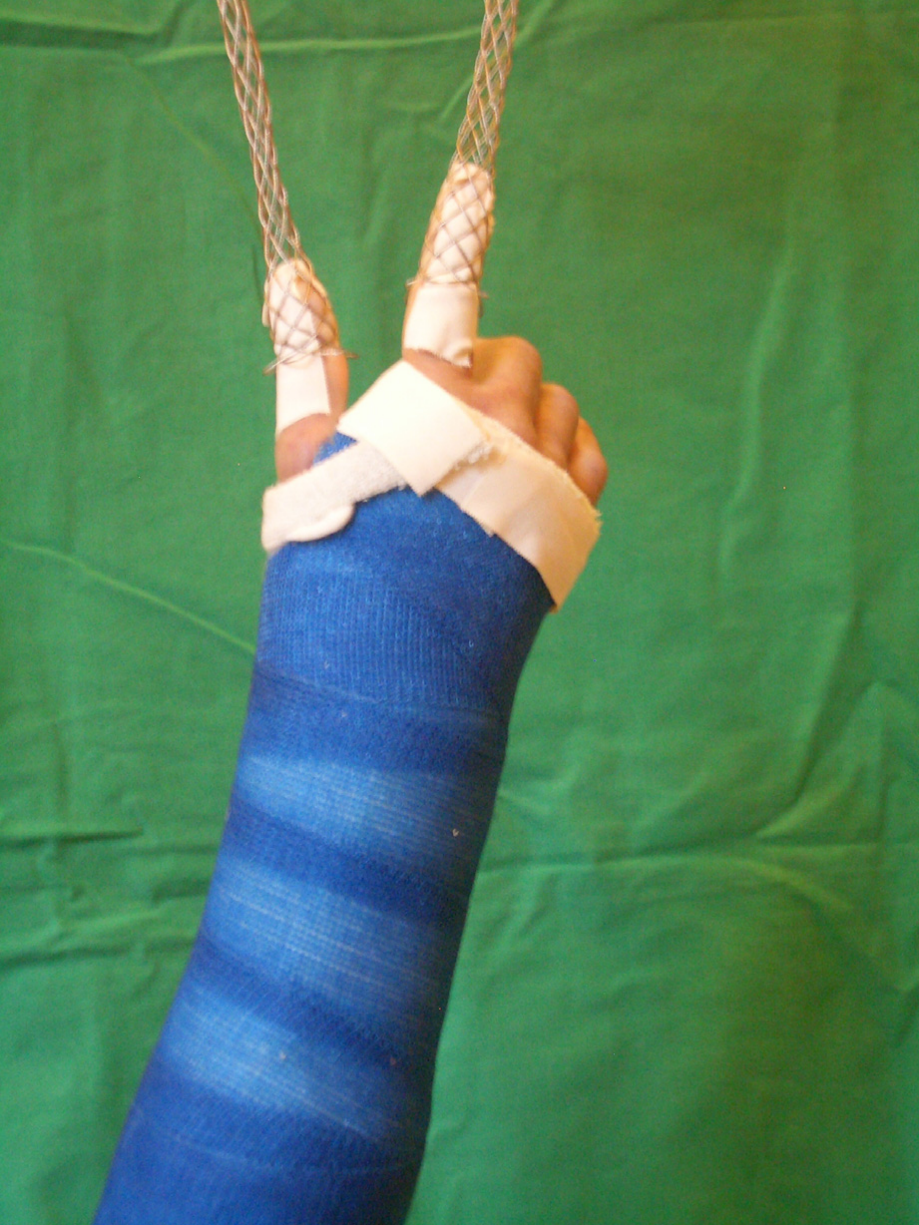
**Closed reduction and casting**. Closed reduction and casting of an intraarticular distal radius fracture in the finger-trap traction technique with a light cast.

**Figure 3:**
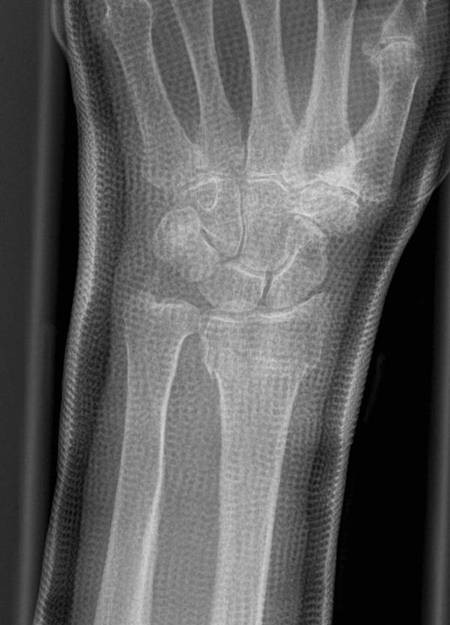
**Post-reduction radiograph of the wrist**. Mild shortening of radial length in the anteroposterior view after casting

All fractures will initially be reduced under image-intensifier control, followed by plaster cast stabilization (Figure 2 and 3). The intervention is performed by the surgeon on charge with proven expertise in reduction and casting maneuvers. Centers may follow their preferred reduction and casting techniques and locally established interventions for pain management (i.e., fracture block or intravenous pain medication).

#### Non-operative treatment

If randomized to non-operative care, cast treatment will be continued for six weeks. Once swelling has decreased, the splint cast is converted into a closed cast (hard plaster or light cast). Patients in the non-operative arm are usually treated in an ambulatory setting.

#### Operative treatment

If randomized to operative treatment, patients will be admitted to the hospital and operated on as early as possible, depending on local tissue conditions, hematoma and swelling and suitability of the patient for anaesthesia.

**Figure 4:**
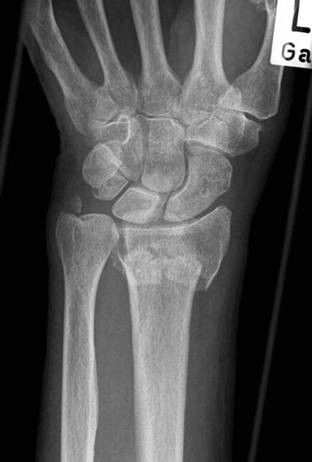
**Intraarticular distal radius fracture**. Anteroposterior radiograph of an intraarticular distal radius fracture type C1-AO/ASIF

**Figure 5:**
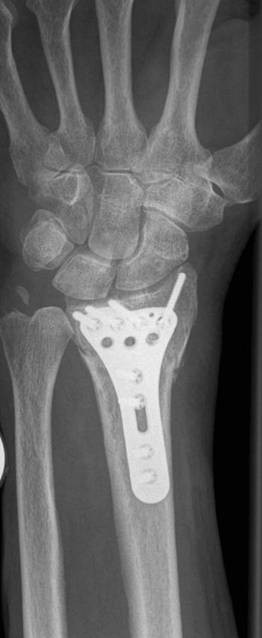
**Operative treatment**. Postoperative radiograph shows restoration of radial length

**Figure 6:**
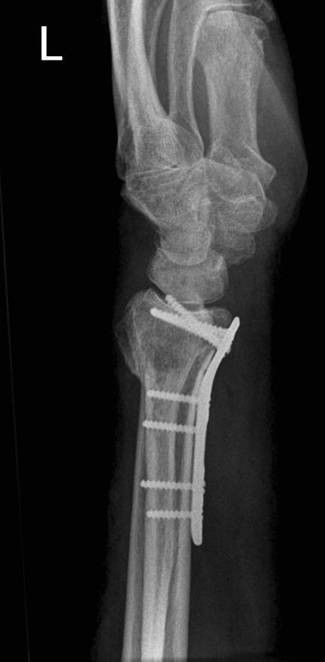
**Operative treatment**. Lateral wrist radiograph shows restoration of palmar inclination

ORIF will be performed via a volar approach and using angle stable locking plates (Figure 4, 5 and 6). The protocol does not mandate certain hardware. ORIF is performed under plexus or general anesthesia and fluoroscopic guidance. All surgeons performing the operation have proven expertise with the procedure and are familiar with the used implants. Post-operative cast stabilization of the wrist may not exceed two weeks.

### Outcome measures

#### Primary outcome

The primary objective of ORCHID is to compare patient-reported outcomes like function, independence and health-related quality of life, measured with the Short Form 36 (SF-36) Physical Component Score (PCS), after operative and non-operative treatment of comminuted wrist fractures rather than surrogate measures (i.e., radiographic healing).

The Short Form 36 (SF-36) is the most widely used generic health assessment questionnaire worldwide. Its validity and suitability to describe wellbeing in a broad range of clinical conditions from a patient's point of view has been studied extensively[41-44].

#### Secondary outcomes

Another quick and validated tool is the EuroQol-5D (EQ-5D). Health utility measures can easily be derived from this instrument, allowing for later health-economic analyses. With the EuroQol-5D the outcomes of the two treatment arms can be compared and health-related quality of life can be measured.

In addition to generic forms, the 30-item, self-reported Disability of the Arm, Shoulder and Hand Score (DASH) questionnaire will be employed as a disease-specific outcome measure.

The DASH was selected as the preferred specific outcome tool because of the longer experience gained with this instrument in Germany. Cross-cultural validation started as early as 2003 [45]. DASH is the recommended tool for outcome assessment in the national clinical guideline for managing fractures of the distal radius http://www.uni-duesseldorf.de/AWMF/ll/012-015.htm and shows comparable psychometric properties to the Patient-Rated Wrist Evaluation PRWE in evaluating recovery after a distal radius fracture[46].

The SF-36, the DASH and the EuroQol-5D are assessed as patient completed questionnaires with assistance of a study nurse during the stay in the clinic or at the outpatient visit.

Wrist function in terms of range of motion (ROM) will be measured using a goniometer. Finally, we will address the patients' independence and ability to cope with everyday activities, nursing home admissions and the need for assistance in activities of daily living, and mortality. Complications (e.g., loss of reduction, hardware failure and misplacement, wound healing problems, infections, revisions, neurologic impairment, pressure ulcera and others) as well as serious adverse events (SAE) will be recorded throughout the study, and regularly evaluated by a Data Safety Monitoring Board (DSMB).

### Evaluation and follow-up

Clinical evaluation and trial documentation consists of six visits: baseline (V1), intervention (V2) and four postoperative follow-up examinations ending with V6 after 12 months (see summary in Table 1).

**Table 1:** Summary of trial-specific interventions and documentation

	V1	V2	V3	V4	V5	V6
	Baseline	Treatment	1 d post-intervention	2 w post-intervention	3 mo post-intervention	12 mo final F/U
Assessment	LI	LI, RA	LI, RA, SR	LI, RA, SR	LI, RA, SR	LI, RA, SR
Entry criteria	X					
Randomization	X					
Radiographs	X	X	X	X	X	optional
SF-36					X	X
EQ-5D			X		X	X
DASH					X	X
Wrist ROM					X	X
Complications		X	X	X	X	X
SAE		X	X	X	X	X

LI = local investigator, RA = research assistant/study nurse, SR = patient self report

#### V1 - baseline examination and randomization

After radiological verification of the suitable fracture type, eligibility criteria are checked at the baseline and trial entry visit. The trial aims and procedures are explained to patients by the responsible local investigators using a leaflet. Informed consent is obtained, and the trial participant is centrally randomized to operative or conservative management. Baseline demographics, comorbidity and medication history of falls and fractures, fear of falling and the diagnosis of osteoporosis are recorded at this time. Falls, fracture falls, and fear of falling are variables associated with a generally poorer prognosis including higher mortality, and predictors of future falls in frail elderly patients [47-50].

#### V2 - treatment

This visit is intended to describe the individual procedures and procedure-related complications (see interventions). In both treatment arms the intervention is performed by the responsible surgeon.

#### V3 - one to six days post intervention

Radiographs are taken to evaluate the quality of reduction (radial inclination, palmar inclination, ulnar length, as well as the appropriate positioning of hardware in the surgical trial arm). To gain information on self-rated independence and wellbeing prior to the fracture event, the EuroQol-5D will be queried at this time. The investigators are aware of the limitations of retrospective assessment of quality of life but consider a baseline rating important for longitudinal comparisons. To avoid bias caused by discomfort and pain in the acute fracture setting, baseline information is gathered after fracture stabilization and sufficient pain control.

#### V4 - two weeks (± 3 days) post intervention

The two-week control is crucial for deciding whether conservative treatment is likely to lead to bony consolidation in an acceptable anatomical position, or whether conversion to surgical fixation is deemed necessary. Between V2 and V4, another attempt of close reduction and casting is allowed. The duration of hospital stay, complications and SAE, and concomitant (pain) medication are recorded.

#### V5 - three months (± 7 days) post intervention

Patients are seen in an outpatient visit and are examined by the responsible surgeon. Wrist radiographs are obtained after three months, and will be scrutinized for healing and alignment. Apart from other local complications, signs of chronic regional pain syndrome (CRPS I) are assessed and the number of physiotherapy courses are documented.

The SF-36, the EQ-5D, the DASH and wrist range of motion will be assessed by a trained study nurse to limit bias.

#### V6 - twelve months (± 1 month) post intervention, final follow-up

Patients are invited for an outpatient visit and are examined by the responsible surgeon for the final follow up. Radiographs of the wrist are obtained if deemed necessary by the surgeon. Further outcomes collected by a study nurse comprise the the SF-36, the EQ-5D, the DASH, and wrist ROM as well as complications and SAE.

If patients are not able to visit the study center, a home visit is done by a trained study nurse, assessing all scores and performing the physcial examination.

**Figure 7:**
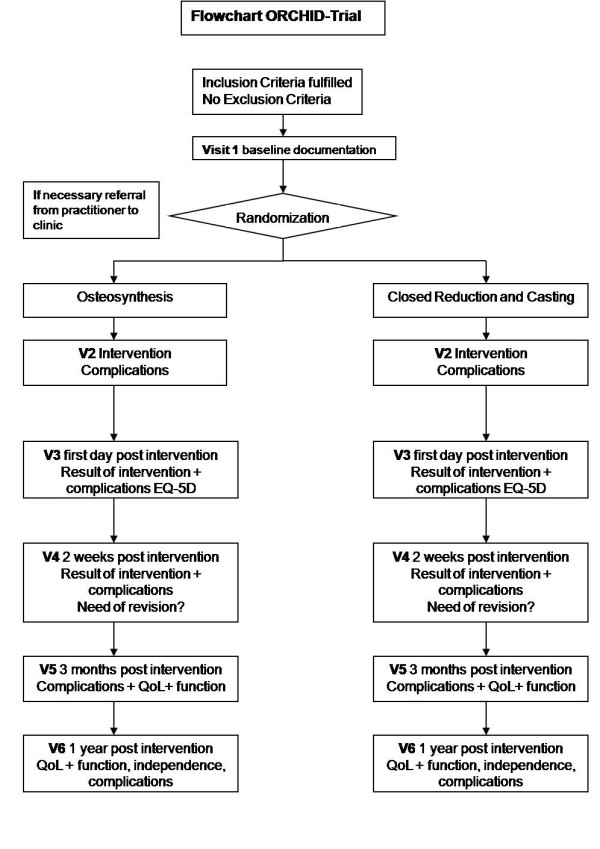
**CONSORT flowchart diagram**. Frequency and scope of study visits

Figure 7 shows the frequency and scope of the study visits.

### Blinding

Options for blinding outcome-assessors were discussed extensively by the members of the trial steering committee and methodological advisory board. The panel members consented to no blinding, considering that blinding of a non-operative treatment (casting) against a surgical intervention is rather complex and would neccessitate time, effort and expenditures. The SF-36 will be assessed by an independent examiner after three months and one year, limiting the degree of observer bias for this endpoint.

### Statistical analysis

#### Sample size and power calculation

The sample size is based on the primary outcome variable and the primary analysis. In the German branch of the International Quality of Life Assessment (IQOLA) Project, the linear T-score transformed, US factor weighted PCS of the SF-36 score from 2525 subjects showed a mean of 50.6 with a standard deviation of 9.8[43]. Standard deviations of about 10 have been observed in studies of elbow fractures as well [51].

Different assumptions can be found about the minimally clinically relevant differences (MCRD) in SF-36 scales. The ORCHID investigators consider an MRCD of 8 points with a standard deviation of 30 clinically relevant. For the PCS scale with a standardized mean of 50 and a standard deviation of 10, this results in a MCRD of 8/3 = 2.67 points. This is close to Walters MCRD proposal of a clinically relevant difference of 2.5[52]. According to another statistical approach, the MCRD calculates to 2.83, accounting for a reported reliability coefficient of 0.92. Using a more conservative MCRD of 2.5 with a standard deviation of 10, 2 × 252 = 504 patients are needed to detect this difference with a power of 80% and a two-sided alpha of 5% with a test for means in independent samples.

The trial should also have sufficient power for the sensitivity analyses with the per-protocol-population. Incorporating 15% drop-outs (that is, deceased patients and losses to follow-up), a total sample size of n = 600 patients is needed.

#### Data-analysis

The confirmatory analysis will be performed on the basis of an intention-to-treat (ITT) population and with respect to ITT principles. In this protocol, we adhere to the original meaning of the intent-to-treat analysis, namely analyzing patients according to their random assignment, regardless of the treatment actually applied.

Additional analysis will be conducted on the per-protocol population (e.g., patients assigned to closed reduction but crossing over to ORIF).

Descriptive statistics for continuous variables and scores include the number of non-missing observations, mean, standard deviation, median, minimum and maximum, performed for the different subgroups as well as the treatment groups and overall. The description of categorical variables (ordinal or nominal) includes the number and percentage of patients belonging to the relevant categories in the trial group as well as to each treatment group.

The primary efficacy endpoint is the Physical Component Score (PCS) of the SF-36, ascertained 12 months after randomization. The underlying two sided null-hypothesis is that both interventions lead to similar means of the SF36-PCS in both intervention groups 12 months after randomization.

[1] H_0_: μ1 - μ2 = 0

The alternative hypothesis is that any intervention performs better than the other:

[2] H_A_: μ1 - μ2 ≠ 0

A confirmatory intention to treat analysis (2-sided test), including all patients *as randomized*, will be performed on the mean differences in the SF36-PCS values between the two treatment groups. Analysis of covariance (ANCOVA) techniques will be used to detect possible treatment effects, with trial center, fracture type and gender as factors and age as continuous covariates.

Secondary endpoints will be analyzed in an exploratory fashion, using appropriate statistical methods based on the underlying distribution of the data. Sensitivity analyses will be conducted including different populations (per-protocol population, intention-to-treat population, with values of drop-outs set to worst scores), statistical methods (nonparametric methods, mixed effects analysis [53]), and covariates.

Graphical methods including scatter plots and box-plots will be used to visualize possible correlations between continuous variables and differences between intervention groups. We will explicitly state numerators and denominators in case of incomplete data, and attempts will be made for multiple imputation where sound and necessary. All analyses will employ SAS Version 9.1.

### Patient drop-out and protocol violations

Patients lost to follow-up or who leave the study early are documented, including the reason for drop-out. Deviations from the protocol-driven surgical technique, e.g. dorsal plating or K-wire pinning will be recorded. Patients who cross-over from one treatment arm to another, e.g., those having been assigned to casting but undergoing secondary ORIF due to unacceptable loss of reduction, will be evaluated according to ITT.

### Safety

All adverse events (AE) during the study period will be documented. Rates of complication and serious adverse events (SAE) are part of the secondary endpoint analysis and will be closely monitored. Expected complications in both intervention groups include loss of reduction, hardware displacement, hardware failure, sensomotoric deficits, carpal tunnel syndrome, wound healing problems, superficial and deep infection, skin pressure ulcera, malunion, nonunion, revision surgery, and CRPS.

The severity of complications is categorized into minor and major and their causality to the trial intervention is evaluated. Complications leading to surgical revision, clinically important morbidity or mortality are classified as SAE. All SAE occurring during the study period (regardless of their association with the trial intervention) are graded according to their magnitude and their outcomes.

A Data Safety and Monitoring Board (DSMB) was constituted consisting of independent trauma surgeons and a statistician. Annually, the board members are furnished with safety data (frequency and distribution of SAE) and will provide recommendations to the principal investigator and the steering committee to decide about continuation or modification of the trial.

### Trial documentation and data collection

Paper based case report forms (CRF) will be used for documentation. Entries will be transferred into an electronic format by trained personnel, using a double-data-entry strategy. All source documents are available in electronic and paper format.

All complications and SAE are documented on a special documentation sheet. SAE will be reported to the principal investigator as soon as possible by fax, at the latest within five days.

### Monitoring

To ensure patients' safety and integrity of clinical data, e.g., correct informed consent procedure and primary endpoint evaluation in adherence to the study protocol, continuous clinical monitoring procedures are set in place. Clinical monitors from the Coordination Centre for Clinical Trials (KKS), Heidelberg, Germany, will introduce all sites to study procedures and documentation before beginning of the trial. Onsite monitoring during the study will be done by personal visits according to the standard operating procedures of the KKS. The monitors review amongst others the entries into case report forms on the basis of source documents. Therefore, each investigator must allow monitors full access to all essential documents, and must provide necessary support to the monitors. In addition to regular site visits, continuous support is guaranteed through email- and phone communication between monitor and trial site.

### Patient data safety

Throughout the trial, subjects will be identified solely by an individual identification code. Case report forms will be stored in accordance with local data protection laws and will be handled with strictest confidence. The appropriate regulations of local data legislation will be fulfilled in their entirety. Authorized persons only (e.g., clinical monitors) will have access to personal data. During onsite visits, clinical monitors will inspect subject-related data to ensure adherence to data protection laws and correctness and completeness of data.

### Ethical issues

The procedures set out in the trial protocol, pertaining to the conduct, evaluation, and documentation of this trial, are designed to ensure that all persons involved in the trial abide by Good Clinical Practice (GCP) [54] and the ethical principles described in the Declaration of Helsinki [55]. The trial is carried out in compliance with local legal and regulatory requirements, although German Drug Law and Medical Device Law are not applicable.

Before the first subject was enrolled, all ethical and legal requirements were met by inclusive unreserved approval by institutional review boards (IRB). The final study protocol and the final version of the written informed consent form were approved by the ethics committee of the University of Ulm, who is responsible for the Principal Investigator. The critical assessments of risks and benefits have been performed by medical experts in advance.

Each site's principal investigator ensures that all persons assisting with the trial are adequately informed about the protocol, the trial treatments, and their trial-related duties and functions.

Before being enrolled onto the clinical trial, each subject must consent to participate after nature, scope, and possible consequences of the clinical trial have been explained to him or her in an understandable oral and written form. The subject must give consent in writing or orally in presence of an independent witness before randomization.

Results of study will be published according to the recommendations issued by the Enhancing Quality and Transparency of Health Research (EQUATOR) [56] network, specifically the most recent version of the Consolidated Standards of Reporting Trials (CONSORT) statement.

## Discussion

Fractures of the distal radius are an emerging public health concern. With the reversal of the aging pyramid, an epidemic of these functional important injuries to the wrist and hand are projected, that need to be managed at institutions of different levels of care in the industrial countries.

The invention of locking plates that show excellent stability even in the osteoporotic bone increased the rate of surgical procedures for fractures that had rather been managed conservatively in the past. It is, however, unclear whether the known biomechanical advantages translate into better clinical outcomes. In the US, it could recently been shown that the choice of treatment depends on age, the place of living, insurance and other issues unrelated to fracture severity and grading[57].

Current evidence, specifically reliable information from RCTs, is insufficient to counsel patients and their relatives with regard to the most convenient, efficient, and safe method to restore function, activity, grip strength and so on after a complex distal radial fracture.

As far as we are aware, the ORCHID trial is the first multicenter RCT that aims at generating conclusive evidence for clinical practice guidelines and health-care policies on the most appropriate way of treating intraarticular distal radius fractures.

## Research plan

Start date: September 2008

## Abbreviations

AE: adverse event; ANCOVA: analysis of covariance; AO: Arbeitsgemeinschaft Osteosynthese; BMI: Body-Mass-Index; CRC: closed reduction and casting; CRPS I: chronic regional pain syndrome type I; CRF: case report form; DASH score: disability of the arm, shoulder and hand score; DSMB: data safety monitoring board; GCP: good clinical practice; IQOLA: International Quality of Life Assessment; ITT: intention to treat; IRB: institutional review board; MCRD: minimally clinically relevant difference; MCID: minimum clinically important difference; NSAID: non-steroidal anti-inflammatory drug; ORCHID: open reduction and internal fixation versus casting for highly comminuted intra-articular fractures of the distal radius; PCS: physical component score; ORIF: open reduction and internal fixation; RCT: randomised controlled trial; ROM: range of motion; SAE: serious adverse event; SF-36: ShortForm 36 score; SF-36-PCS: ShortForm 36 - Physical Component Score

## Competing interests

The authors declare that they have no competing interests.

## Authors' contributions

FG conceived the trial, secured trial funding and is the principal investigator of the trial. CB contributed to trial design, developed the trial protocol, drafted the manuscript and acts as coordinating investigator of the trial. DS developed the trial design and the trial protocol. TB coordinates the statistical analysis. IR and CS participated in development of the trial protocol and coordinates multi-center management. SL participated in development of the trial protocol and coordinates trial monitoring. All authors contributed in development of the trial protocol and the CRF. All authors contributed to the manuscript and approved the final version of the manuscript.
